# Toxicity and Apoptosis Related Effects of Benzimidazo [3,2-α] Quinolinium Salts Upon Human Lymphoma Cells

**DOI:** 10.2174/1874104501711010054

**Published:** 2017-06-30

**Authors:** Christian Vélez, Jessica Soto, Karoline Ríos, Luz Silva, Wigberto Hernandez, Luis A. Rivera, Ana I. Ortiz-Colón, Osvaldo Cox, Beatriz Zayas

**Affiliations:** 1 Universidad Metropolitana, School of Environmental Affairs, San Juan, Puerto Rico; 2 Institute of Biomedical and Forensic Sciences Research of Puerto Rico, San Juan, Puerto Rico; 3 Department of Chemistry University of Puerto Rico, San Juan, Puerto Rico; 4 Department of Chemistry, University of Puerto Rico at Mayaguez, San Juan, Puerto Rico; 5 Department of Anatomy and Neurobiology, University of Puerto Rico School of Medicine, San Juan, Puerto Rico

**Keywords:** Benzimidazo[3,2-*a*]quinolinium Salts, BQS, Apotosis, Apoptosis Inducing Factor, Anti cancer, Unnatural alkaloid, Diffuse large B-cell Lymphoma

## Abstract

**Objectives:**

The present study evaluates novel cationic quinoline derivatives known as benzimidazo[3,2-*a*]quinolinium salts (BQS) named NBQ-48 and ABQ-48 that have structural similarities to known anti-cancer substances such as ellipticine and berberine.

**Methods:**

Toledo human lymphoma (ATCC CRL2631) cells were treated for 24 to 48 hours. Apoptosis related endpoints such as cell cycle arrest, mitochondrial damage, RNS and ROS generation and the activity of several apoptosis related proteins such as caspases and apoptosis inducing factor (AIF) were studied using fluorescence staining and western blot respectively.

**Results:**

Results indicated a higher toxicity from the amino substituted ABQ-48 versus the NBQ-48 (GI50’s of 50uM versus 100uM respectively). Both compounds induced cell death through various apoptosis related endpoints including a decrease in mitochondrial membrane potential with an increase in ROS and activation of the effector caspase 3. Interestingly, AIF release was observed on cells treated with the amino substituted ABQ-48 but not on the nitro substituted NBQ-48 samples suggesting a caspase independent mechanism for ABQ-48.

**Conclusions:**

The results obtained presents the toxic effects of two novel benzimidazo[3,2-*a*]quinolinium salts in human lymphoma tumor cells. The identified mechanism of action includes multiple apoptosis related effects. Furthermore the data presents a clear variation in caspase dependent or independent mechanism for each compound.

## INTRODUCTION

Diffuse large B-cell lymphoma is one of the most prevalent Non-Hodgkin’s lymphomas (NHL) in the United States [1]. This disease has been considered as one of the most common NHLs in the developed world. Treatment has been standardized using the cyclophosphamide, doxorubicin, vincristine and prednisone (CHOP) regimen [2] alone or in combination with monoclonal antibodies however relapse can occur. The search for novel therapeutics has uncovered many promising substances that can be used along with these established treatment regimes, however none of the proposed regimes have absolute success rate.

Among these possible alternatives are water soluble cationic compounds such as quinolines or their derivatives. Quinoline drugs have been used with success on human cancers of the blood [3]. In this study, we present a pair of unnatural alkaloids known as benzimidazo[3,2-*a*]quinolinium salts (BQS) named 3-nitro- and 3-amino-7 benzylbenzimidazo[3,2-*a*]quinolinium chlorides (NBQ-48 and ABQ-48, respectively). These compounds feature a heteroaromatic planar system which incorporates a quaternized nitrogen Figs. (**1a** and **b**). These novel compounds have a structural similarity with known anti-cancer substances such as ellipticine and berberine. Other members of this compound family have previously shown biological activity against leukemia, Ehrilch ascites and epitheloid carcinoma among other tissues screened at the National Cancer Institute [4-6]. Additional reported activity includes formation of dG – BQS adducts with DNA in an hypoxic environment [7]; this BQS-DNA interaction is similar to what has been reported with ellipticine [8]. These previous studies with nitro-substituted BQS (NBQ) have shown possible DNA mediated mechanism of action, the amino-substituted compounds however have shown greater toxicity and some activity towards blood cancer as described by the NCI screening panel [9]. In this study we assess the cell death mechanism of action of these novel compounds including apoptosis related endpoints such as cell cycle arrest, mitochondrial damage, caspases activation, RNS and ROS generation and the activity of main apoptosis related proteins.

## MATERIAL AND METHODS

### Tested Compounds

The tested compounds NBQ-48 and ABQ-48 are novel compounds synthethized and characterized as described in previously published study [9]. Compounds were prepared in stock solutions of 3mM concentration prepared with sterile, filtered, and deionized water. The stock solutions were kept in sealed glass vials and stored at 4°C to maintained the stability and avoid photo degradation. Biological activity experiments were performed in triplicate.

### Cell Toxicity

The Toledo diffuse large cell non-Hodgkin's B cell lymphoma cell line (ATCC CRL2631) was obtained from American Type Culture Collection (Manassas, VA) and selected for toxicity analysis and biological activity experiments. Cytotoxicity analysis through the concentration that inhibits 50% of cell growth (GI_50_) was determined using trypan blue (0.4%) exclusion (Sigma Aldrich, St. Louis, MO) for a period of 48 hours. Cultures at a density of 5 x 10^5^ cells were grown on 12.5cm^2^ flasks in triplicates before being exposed to the experimental compounds. Samples contained a total volume of 3.5mL including modified RPMI 1640 media (10% FBS) and the tested compound. Cells were exposed to the tested BQ’s at concentrations ranging from 0 to 500µM to determine the GI_50_. Biological activity assays were performed by treating cells with the determined GI_50_ of each tested compounds.

### Mitochondrial Membrane Permeabilization (MMP)

Mitochondrial membrane permeabilization as an indicator of apoptosis induction was analyzed with the Nucleo Counter NC-3000 (Chemometec, Allerød, Denmark) instrument following manufacturer’s instructions and implementing the mitochondrial potential JC-1 assay. This assay is based on a membrane potential accumulation in the mitochondria and is observed by a shift in dye emission from red to green after application of the JC-1 solutions 5,5',6,6'-tetrachloro-1,1',3,3'-tetraethylbenzimidazolocarbocyanine iodide assay (200µg/ml JC-1) and DAPI in PBS(1µg/ml). Cell cultures of 3 x 10^6^ cells/flask were exposed to each of the BQs at their respective doses along with the two positive controls (camptothecin 10µM dose and valinomycin 5 µM) and incubated for 48 hours. Samples were stained with the JC-1 reagent then stained with 1μg/ml DAPI solution, and analyzed immediately. A one way ANOVA and a Post Hoc Test Tukey were performed.

## OXIDATIVE STRESS

### Generation of Reactive Oxygen Species (ROS)

Determination of ROS generation was performed using 2,7-dichlorofluoresce diacetate (DCFH-DA), as described by Gutierrez-Praena *et al*. [10], and Park *et al*. [11]. Intracellular esterases hydrolyze DCFH-DA when it diffuses across cell membrane to a non-fluorescent compound (DCFH). DCFH is quickly oxidized in the presence of ROS to a high fluorescent DCF, which is proportional to the ROS levels. After 24-hour drug treatment, cells were harvested and incubated with 200 µl of 20µM DCFH-DA in medium at 37 ^0^C for 30 min, washed with phosphate buffered saline (PBS), and re-suspended in 200 µl of PBS. ROS production was assessed after treatment in 96-well microplates. Cells were then transferred to a 96-well microplate and analyzed. DCFHDA fluorescent probe converted to DCF reveal ROS levels by green fluorescence at an emission wavelength of 535 nm and excitation wavelength of 485 nm using the fluorostar Optima fluorescence reader (BMG, Ortenberg, Germany). Results (percentage, %) were shown after negative control standarization.

## GENERATION OF REACTIVE NITROGEN SPECIES (RNS)

RNS determination was performed using 2,3-diaminonaphthalene (DAN), according to the method by Misko **et al*.* [12], and Kleinhenz **et al*.* [13] with minor modifications. DAN solution was prepared at 0.05mg/ml in 0.62N HCl. After drug treatment, supernatants were recovered and centrifuged at 2,000 g for 2 minutes. Supernatant aliquots (85 μl) in triplicates were placed into 96-well plates and 10 μl of DAN was added and incubated for 15 min at 37 ^0^C. After 15 min, 5 μl of 2.8N NaOH was added to each well. Analysis of samples were performed using fluorescence excitation at 360nm and emission at 440nm with the Fluorostar Optima fluorescence reader (BMG, Ortenberg, Germany).

## DNA Fragmentation

DNA Fragmentation, was also used to asess apoptosis [14]. This event which is facilitated by nucleases that degrade nucleic acids can be quantified using DNA content measuring cells containing less than 1DNA equivalent (Sub - G_1_). The method for the Nucleo counter NC3000 assay is based on the washing of small DNA fragments and the retention of 4',6-diamidino-2-phenylindole (DAPI) stained fragments of a higher weight. After treatment with BQ’s at previously described conditions, cells are fixed with 70% ethanol, incubated and stained with 1µg/ml DAPI and analyzed by using image analysis measuring DAPI intensity with the Nucleo Counter NC-3000 (Chemometec, Allerød, Denmark). A one way ANOVA with Tukey post test was performed to compare data.

## SDS-PAGE and Western Blot

To determine the activity of Caspase 8, Caspase-3 and apoptosis inducing factor (AIF) western blot was performed as described by Ji **et al*.* [15], and Towbin [16], with few modifications. Following the treatment, lymphoma cells were harvested, the cell lysate was transferred to 1.5ml tubes, homogenized and centrifuge at 12,000 x g for 19 min at 4 ºC. The supernatant was transferred to a fresh tube and protein concentration was determined using Bio-Rad Protein Assay Reagent (Bio-Rad). Equal amount of protein samples was resolved on SDS-PAGE and transferred to 0.2 μm nitrocellulose membranes(Bio-Rad,USA). The membranes were immunoblotted with Caspase 8 associated protein 2, Caspase-3 full length and AIF (Cell Signaling Danvers, MA) antibodies followed by secondary antibodies. The chemiluminescence signals were visualized using Odyssey Western Blot reagents (Licor) and the LiCor Odyssey imaging system (Licor, Lincoln, NE).

## Annexin V Assay

The annexin V assay wideley is used as a tool for the detection of phosphatidylserine (PS) on the surface of cells, a key event in apoptotic cells. Approximately 3x10^6^ cells were treated with the GI_50_ dose of each compound and negative and positive controls (ultra pure water and camptothecin respectively). After cells treatment with the two tested compounds and controls an aliquot of 5x10^5^cells cells from each sample was stained using annexin V – Cy3 conjugate (Biovision,San Francisco USA) as per manufacturer’s instructions. The fluorescence was measured using a Fluorostar Optima fluorescence reader (BMG lab tech, Cary, NC) using the standard 544 - 590 nm filters.

## RESULTS

### Toledo Cell Toxicity

The capacity of the two benzimidazo[3,2-*a*] quinolinium salts (BQS) to inhibit cell viability was assessed after a 48 hour exposure on Toledo cultures. Cells were treated with doses ranging from 0µM to 500µM and showed clear differences in dose response effect on cells treated with the two tested compounds. Table (**1**) presents the GI_50_ doses obtained for each compound. Observed GI_50_ for NBQ 48 and ABQ-48 was 100 µM and 50 µM respectively. The substitution at R_1_ with the amino group appears to result in a higher cell death effect with twice the toxicity as compared to the nitro substituted compound indicating that ABQ-48 (amino substituted) was the more toxic of the two tested compounds. These results suggest a structure – activity variation among both test drugs. This finding is in accordance with our previous work with other cell lines which demonstrated that ABQ-48 has greater biological activity in comparison to its Nitro analog [9].

### MMP

Mitochondrial fragmentation and membrane dysfunction is a common characteristic on apoptotic cells [17]. During JC-1 staining, the dye is incorporated into the mitochondria where it will form green monomers which emit around 527nm or the red aggregates which emit around 590nm. A high mitochondrial transmembrane potential (ΔΨ_m_) will be seen as red stained cells whereas depolarized cells will emit green. By measuring these emission ratios we can measure the loss in membrane potential characteristic of apoptosis. Fig. (**2**) presents the values obtained for each compound indicating the effect on the mitochondria. NBQ 48 presented 51.3% of cells with permeabilized membrane while ABQ-48 showed a 57.67% of cells afected. Both compounds were compared with the positive controls camptothecin (51.6%) and Valinomycin (55.3%). All samples exposed to the tested compounds presented statistically significant diference in the permeabilization of mitochondria (P<0.05) in comparisson to the negative control .

### ROS

Impairment of mitochondria function at high levels of ROS has been a well established event [18, 19]. In our study Lymphoma B-cells exposed to NBQ-48 and ABQ-48, at concentrations described above, showed significant increase in the ROS content Fig. (**3**), when compared to the negative control. These ROS levels in treated cells with NBQ-48 and ABQ-48, are similar to the positive controls (camptothecin and cisplatin). These findings support the observed MMP indicating altered mitochondrial potential due to exposute to the tested BQs. Apoptosis mechanisms by high levels of ROS characteristically involve receptor activation, mitochondrial dysfunction and caspase activation proteins such as the Bcl-2 family have been related to this process. Prior studies presented loss of mitochondrial membrane potential (MMP) in BQ compounds, which in turn triggered the apoptotic process. Augmented generation of superoxide anions impairs cell function and has been regarded as an important event in the cell death mechanism [20].

### RNS

Total RNS production assessment of tested drugs, which includes nitrite and nitrate species, was performed using DAN assay. Results indicate that B-lymphoma cells exposed to ABQ-48 and camptothecin showed significant (p <0.0001) generation of RNS Fig. (**4**), when compared with the negative control. In cells treated with NBQ-48 and cisplatin; catalytic activity coincides with basal level production of nitrite and nitrate species. Disproportionate production of reactive species affects biochemical processes, which include cellular signaling, proteins and DNA. Reactive species (ROS/RNS) include a diversity of molecules, such as superoxide anion, hydrogen peroxide, hydroxyl radical, and peroxynitrite [21-23]. Production of ROS/RNS could affect processes such as enzymatic systems (example, NADPH oxidases (NOX1) and oxygen reduction in the electron transport chain.

### DNA Fragmentation

**Fig. (5):**
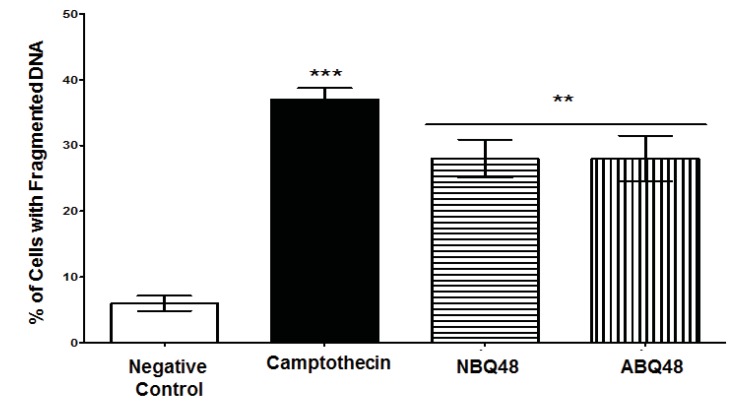
DNA Fragmentation were determined after 24 h of exposure to negative control (vehicle), NBQ-48 (100 µM), ABQ-48 (50 µM), and camptothecin (CP, positive control, 50 µM). Cells treated with: both NBQ-48 and ABQ-48 showed 28% percent of fragmented DNA and CP presented a 37% cells with fragmented DNA. ANOVA Statistical analisis showed significant (p <0.001) difference in cells exposed to CP, ABQ-48 and NBQ-48 when compared with the negative control. Experiment was performed in replicates and normalized with negative control (expressed in percent %). Statistical analysis performed was a one-way ANOVA, with Tukey post hoc test, where p <0.05 was considered to be significant. (P summary;*** = p <0.0001, ** p<0.001).

DNA fragmentation as a marker of apoptosis due to exposure to BQS compounds was measured. Both experimental compounds presented an average value of 28% of cells with fragmented DNA Fig. (**5**). Negative (vehicle) and positive (camptothecin) controls exposed cells presented DNA fragmentataion values of 6% and 37% respectively. Figure 5 presents the percentage of cells with fragmented DNA. Both compounds presented statistically significant DNA fragmentation (P<0.05) comparable to the camptothecin positive control.

### SDS-PAGE and Western Blot

To assess whether ABQ-48 and NBQ-48 induced apoptosis were caspase-dependent, the level of the initiator (caspase 8), effector (Caspase-3) and AIF was analyzed by western blotting using specific antibodies. Table (**2**) sums the results of all tested parameters for both compounds. Protein levels were determined after 24h exposure to negative control (vehicle), NBQ-48 (100 µM), ABQ-48 (50 µM), and camptothecin (CP, positive control, 50 µM). Equal amounts of protein (30 µg) were separated by SDS-PAGE, using the indicated antibodies, B-actin expression was used as a loading control. A specific band was detected for AIF at approximately 57 kDa (as indicated in Fig. (**6**). Caspase 8 was not detected in a significant manner other than in the positive control. Expresion of caspase 3 was detected, significant changes were observed in Caspase-3 antibody endogenous levels of full length caspase-3 (35 kDa) and the large fragments of caspase-3 resulting from cleavage (17 and 19 kDa).

### Annexin V Assay

Results from the applied annexin assay indicated that both compounds presented apoptotic activity at the tested doses. As presented in Fig. (** 7**), the nitro containing BQS (NBQ48) presented a slightly higher apoptotic activity in comparison with the Amino containing (ABQ48) with averages of 28085 versus 25765 Fluorescence standard units (FSU). The highest apoptotic percentage was presented by the positive control camptothecin with 35260 FSU, the lowest apoptosis was observed in the negative control with 13753 FSU. A multiple comparison statistical significance test presented significant difference (P<0.05) of all experimental samples when compared to the negative control indicating that the tested NBQ compounds as well as the positive control induce apoptosis as indicated by PS migration to the outer cell membrane.

## DISCUSSION

This study presents the comparison of two novel benzazolo quinolinium salts, an amino and nitro substituted variants. Although toxicity is significantly higher (GI_50_ of 50uM) in cells treated with the amino substituted ABQ-48 versus the NBQ-48 (GI_50_ of 100uM), both compounds presented similar apoptosis activity as indicated by annexin assay. Our results indicate that both compounds induce cell death through various apoptosis related endpoints. Among these events, a decrease in mitochondrial membrane potential with an increase in ROS and effector caspase 3 activation and PS migration was observed for both compounds. Interestingly, AIF activation was observed on the amino substituted ABQ-48 treated samples but not on the NBQ-48 treated cells which can suggest a caspase independent mechanism for ABQ-48. This result can be linked to the positive detection of RNS in the ABQ-48 samples. It has been documented that RNS production is related to caspase independent apoptosis such as the case mediated by AIF [24, 25].

Our data suggest an intrinsic apoptosis mechanism as indicated by the mitochondrial permeabilization and the absence of caspase 8 activation. In this setting, the permeabilization of mitochondria can lead to the production of ROS, release of caspase activators and caspase independent effectors such as AIF [26]. The fact that our results include positive activity on caspase 3 activation and DNA fragmentation combined with the mitochondria dependent effects suggest that more than one cell death pathway can be activated. It has been documented that AIF and caspase activation can ocurr even in a caspase independent pathway, indeed several studies have established the existence of a caspase 3 feedback loops. In this case downstream caspases can increase AIF release through a positive amplification loop [27]. Compounds derived from ellipticine (structural analogs of the BQS under study), have shown to induce more than one cell death mechanism which can explain why these different endpoints are detected simultaneously [28-30].

Previous work by Zayas *et al*, 2007 [31] on nitro substituted compounds from this family of BQS shows the formation of DNA adducts mediated by enzymatic reduction which suggests one possible cause for the caspase dependent apoptotic cell death of NBQ-48. Enzymatic reduction and adduct formation of ellipticine like compounds has been described as a promoter of apoptotic cell death characterized by events such as DNA damage and inhibition of topoisomerase II leading to classic apoptosis pathways involving caspase activation and mitochondrial dysfunction [32]. Additional work on similar ellipticine analogs has shown that these types of compounds can increase ROS levels and activate AIF translocation in DNA damage apoptosis pathways, [33,34] and that variations in activity among nitro or amino substituted compounds have been observed to change biological activity even among closely related compounds [35,36].

## CONCLUSION

In conclusion, our work expands on previous efforts to describe the biological interactions and cellular effects of the BQS compounds upon lymphoma cells. The results obtained indicate that apoptosis as part of the mechanisms of action is activated in accordance with literature for these type of ellipticine analogs. Mitocondrial membrane potential effects as well as the generation of ROS and RNS and DNA fragmentataion are observed. Furthermore the data present a possible variation in caspase dependent or independent mechanism for each compound Fig. (**8**). Further studies to examine key protein expression and effectors such as Bax, Bcl-2 among others should allow better understanding of the details and differences among the mechanism of action of both types of compounds and members of the NBQ family.

## Figures and Tables

**Fig. (1a):**
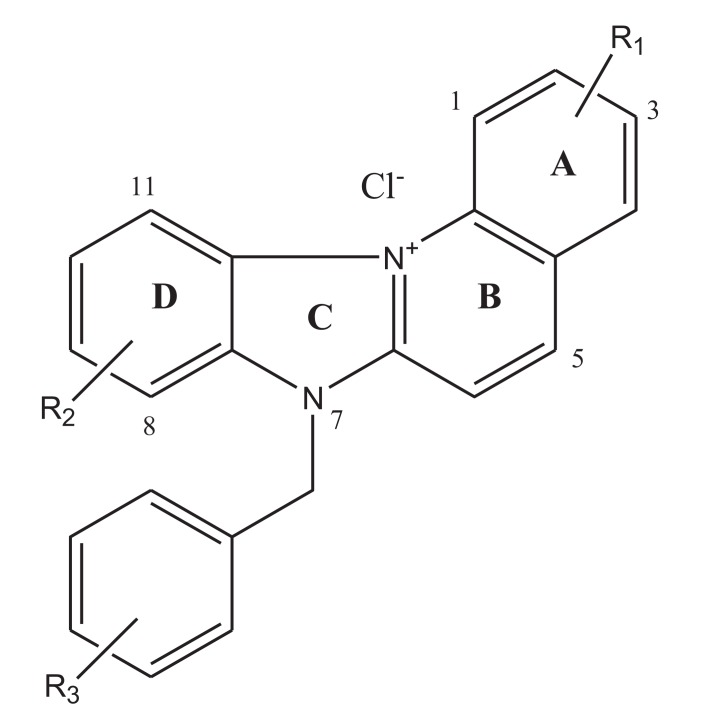
General Structure of the two benzimidazo[3,2-*a*]quinolinium salts studied.

**Fig. (1b):**
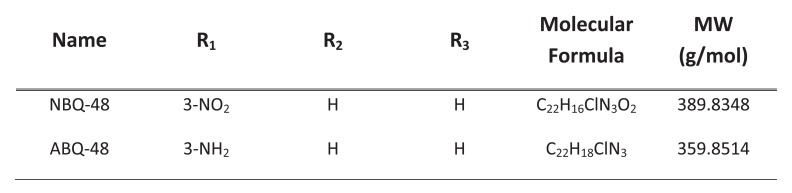
**Molecular formula and R1 susbtituents** ofstudied compounds. NBQ-48 presents a nitro substituted moiety at the R1 position whereas ABQ-48 has an amino group.

**Fig. (2):**
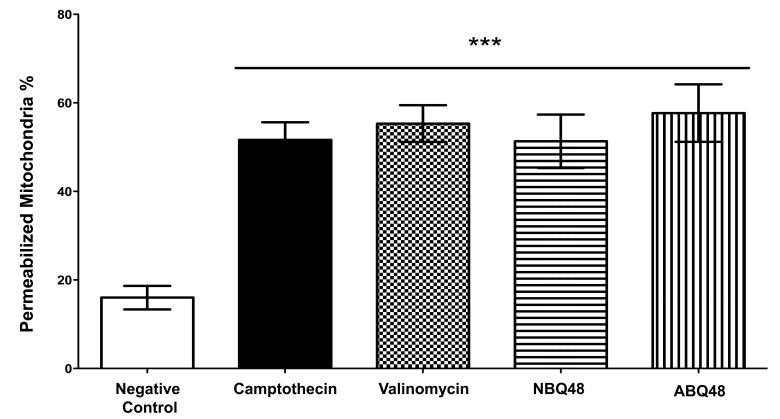
Mitochondrial membrane permeabilization. Results present permeabilization of cells exposed to NBQ-48 and ABQ-48. A 51.3% of Cells treated with NBQ-48 presented permeabilized membrane versus 57.67% of ABQ-48 treated cells presented mitochondrial membrane permeabilization. Positive controls Camptothecin and Valinomycin presented 51.6% and 55.3% respectively. Clear and significant statistical difference was observed on mitochondrial membrane permeabilization among the tested compounds and the negative control (P<0.05).

**Fig. (3):**
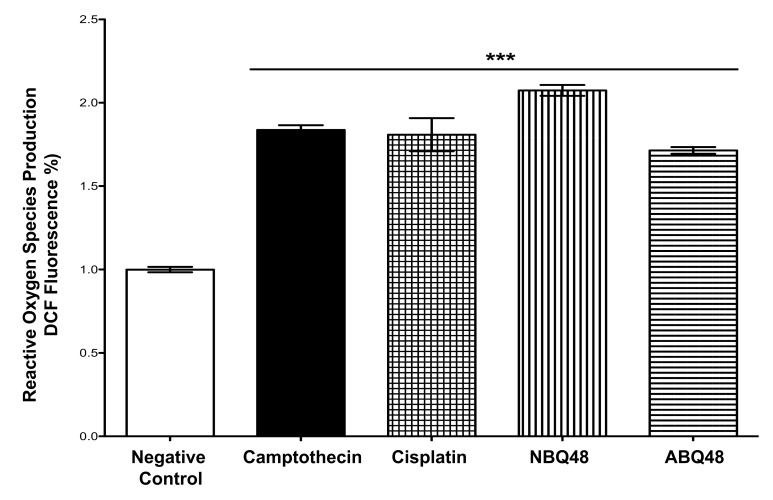
ROS levels were determined after 24h exposure to the negative control (vehicle), NBQ-48 (100 µM), ABQ-48 (50 µM), camptothecin (CP, positive control, 50 µM), and Cisplatin (CS, positive control, 40 µM). Cells exposed to ABQ-48 and NBQ-48 showed significant (p<0.0001) increased ROS levels in comparison to negative control as determined by ANOVA. Statistical analyses performed were a one-way ANOVA, with Tukey post hoc test, where p <0.05 was considered to be significant. P summary; significantly higher in contrast to the negative control (p <0.0001).

**Fig. (4):**
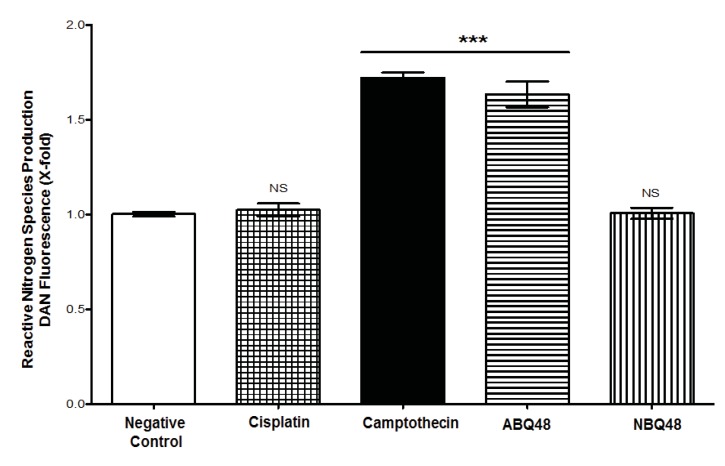
RNS production after 24 h of exposure to vehicle (negative control), ABQ-48 (50 µM), NBQ-48 (100 µM), camptothecin (PC, positive control, 50 µM) and Cisplatin (CS, positive control, 40 µM) was determined. Cells exposed to ABQ-48, and PC exhibited significant induction of reactive nitrogen species (RNS) production on CRL2631 cells when compared to the negative control. Statistical analysis performed was one way ANOVA, with Tukey post hoc test, where p <0.05 was considered to be significant. P summary; significantly different from negative control group (p <0.0001), ns = no significant difference from negative control group (p > 0.05). Results percent (%) was calculated by negative control normalization.

**Fig. (6):**
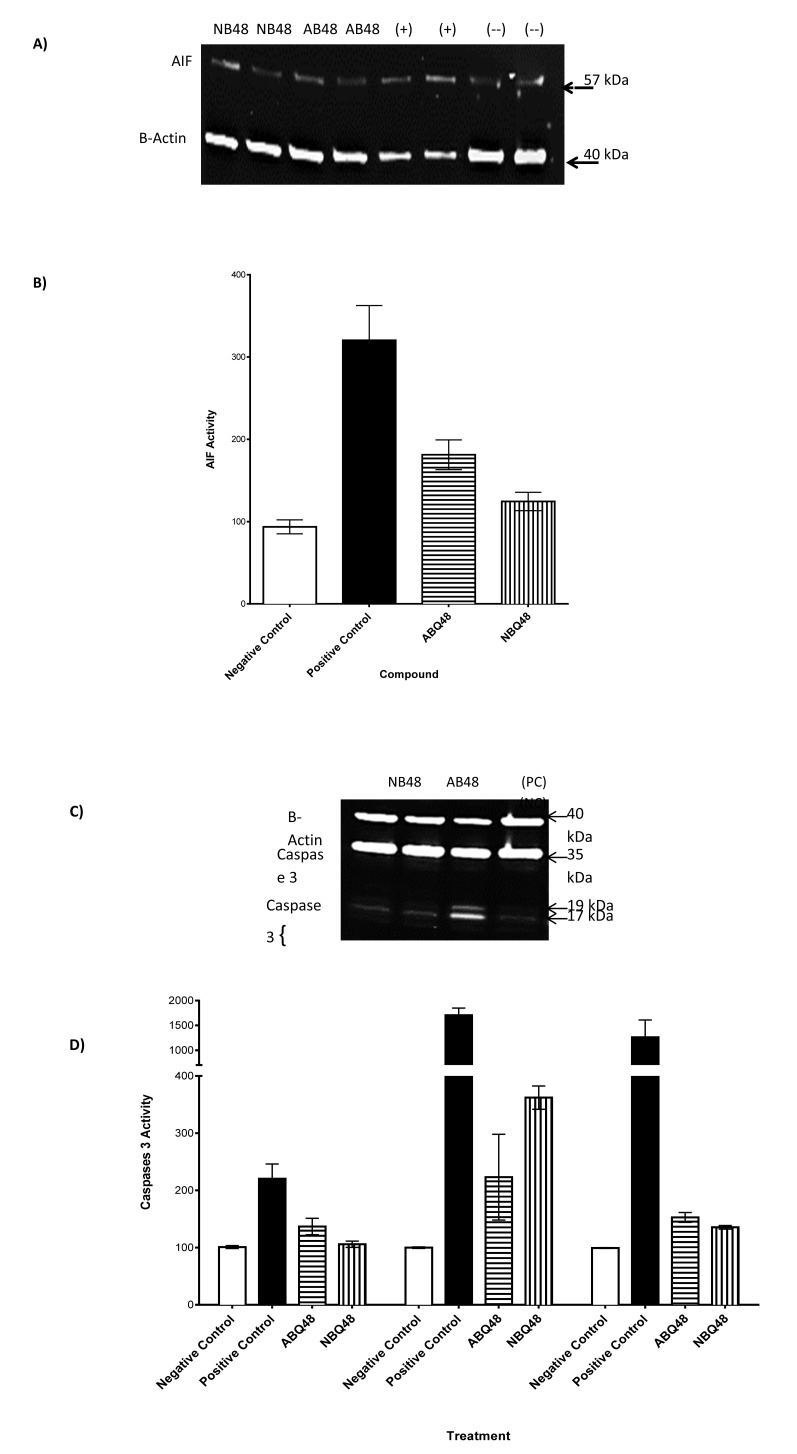
Realease of AIF and activation of caspases after ABQ-48 and NBQ-48 24 hr treatments. (A) Western blot shows lysates of Toledo non-Hodgkin's B cell lymphoma cell line (ATCC CRL2631). A specific band was detected for AIF at approximately 57 kDa (as indicated). (B) At least three independent experiments were performed and data shown are the mean±SD (n=6). ***p < 0.0001 compared to the drugs treated group. (C) Western blot analysis of caspase 3 and 8, results showed presence of caspases 3. (D) Activity of caspase 3 was detected, significant changes were observed in Caspase-3 antibody endogenous levels of full length caspase-3 (35 kDa) and the large fragments of caspase-3 resulting from cleavage (17 and 19 kDa).

**Fig. (7):**
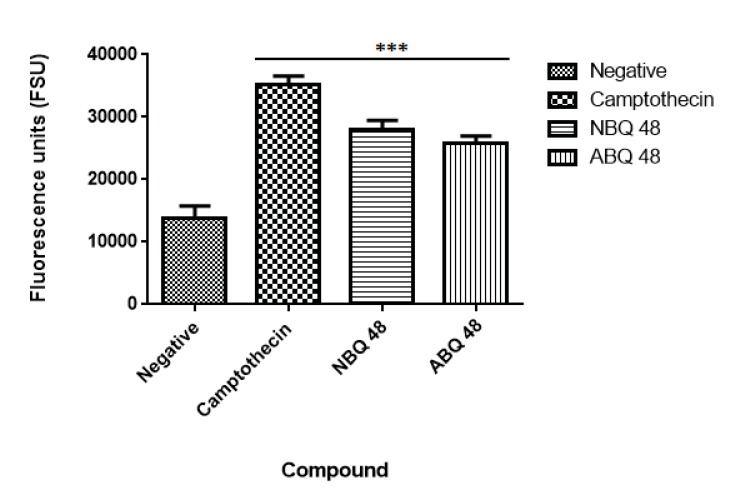
**Annexin V assay**. Negative sample presented the lowest level of apoptosis induction while the highest level was observed in the positive camptothecin control. Both experimental BQ compounds presented apoptosis levels comparable to the positive control. Statistical analysis performed was a one-way ANOVA, with Tukey post hoc test, where p <0.05 was considered to be significant. (P summary;*** = p <0.0001).

**Fig. (8):**
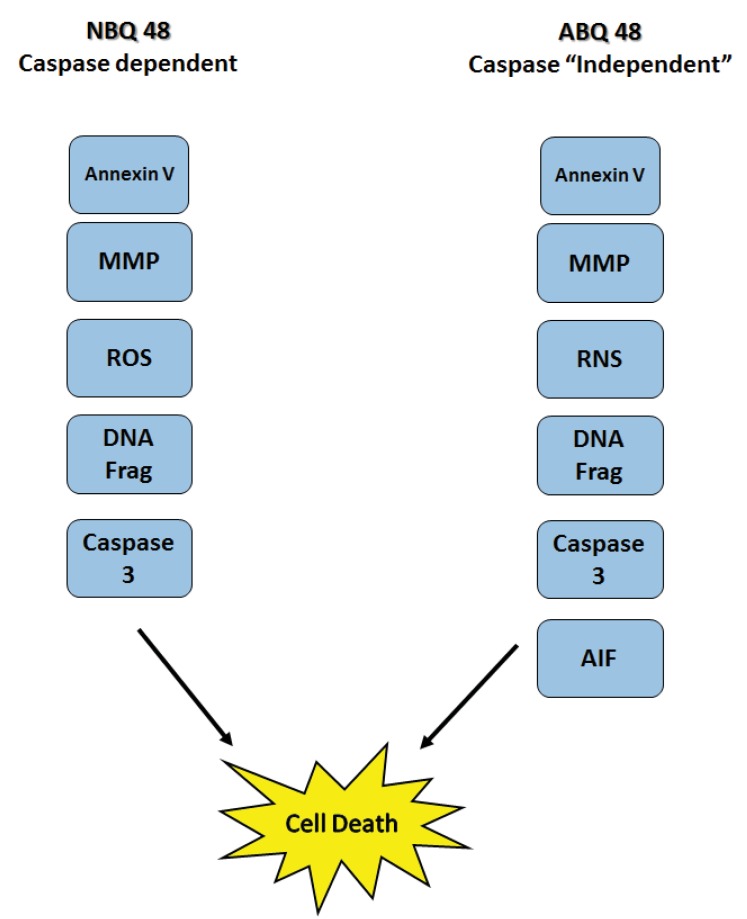
Summary of detected apoptosis related events on Toledo Lymphoma cells after treatment with ABQ-48 and NBQ-48. Results suggest difference in the mechanisms of action. ABQ-48 induced caspase independent with probable downstream caspase activation while NBQ-48 can induce caspase dependent cell death. Mitochondrial membrane permeabilization (MMP), reactive oxygen species (ROS), reactive nitrogen species (RNS), DNA fragmentation (DNA Frag), Apoptosis inducing factor (AIF).

**Table 1:** NBQ-48 and ABQ-48 Growth Inhibition (GI_50_) dose on ymphoma Toledo cells after exposure for 48 hours.

**Antibody**	**NC**	**PC**	**NBQ-48**	**ABQ-48**
Caspase 3 (1:1000)	**-**	**+**	**+**	**+**
Caspase 8 (0.3ugm/ml)	**-**	**+**	**-**	**-**
AIF (1:1000)	**-**	**+**	**-**	**+**

**Table 2:** Western Blot Analysis. Protein samples concentration were adjusted with buffer (80%:20%, Laemmle buffer 5X: b-mercaptoethanol) to 3µg/mL. Antibodies were diluted with Tween OBB 0.1%, probed with goat anti-mouse, goat anti-rabbit (IR-Dye 670 or 800cw labeled secondary antisera, 1:15,000 dilution) in 0.1% Tween OBB for 1 h at room temperature. Washes were repeated three times in 1X PBS-T (0.1% Tween 20). Membranes images were obteined using LiCor Odyssey scanner. (NC= Negative control , PC = Positive control, Experimental NBQ-48 and ABQ-48 compounds.

Antibody	NC	PC	NBQ-48	ABQ-48
Caspase 3 (1:1000)	-	+	+	+
Caspase 8 (0.3ugm/ml)	-	+	-	-
AIF (1:1000)	-	+	-	+
